# Waitlist mortality and recovery among pediatric heart transplant candidates in Germany: A nationwide registry analysis

**DOI:** 10.1016/j.jhlto.2026.100611

**Published:** 2026-06-12

**Authors:** Lisa-Maria Rosenthal, Annemarie Krauß, Felix Berger, Friederike Danne, Oliver Miera

**Affiliations:** a Department of Congenital Heart Disease – Pediatric Cardiology, Deutsches Herzzentrum der Charité (DHZC), Berlin, Germany; b Charité Universitätsmedizin Berlin, corporate member of Freie Universität and Humboldt-Universität zu Berlin, Germany; c Berlin Institute of Health, Berlin, Germany; d German Centre for Cardiovascular Research, Partner Site Berlin, Berlin, Germany

**Keywords:** Pediatric heart transplantation, Waitlist mortality

## Abstract

**Background:**

Clinical outcomes of pediatric heart transplant candidates vary by region due to differences in donor availability and allocation policies. Most registry data originate from North America. We evaluated waitlist mortality, recovery, and associated risk factors among pediatric candidates in Germany.

**Methods:**

All candidates < 18 years listed for heart transplantation in the German Transplant Registry (2006–2020) were retrospectively analyzed. Demographic and clinical predictors of mortality and recovery were assessed.

**Results:**

Among 780 candidates, 441 (56.5%) underwent transplantation, 140 (17.9 %) died, 12 (1.5%) were withdrawn for deterioration, 61 (7.8 %) recovered, 42 (5.4%) were delisted for undocumented reasons, and 84 (10.8%) remained listed. Mean waiting time was 197 days, longest in children 1–10 years. Candidates that died or were removed were younger (median age 3.1 vs. 6.4 years, p=0.005), smaller, and more likely to have congenital heart disease (CHD, 32.2% vs. 21.3%, p<0.001). Waitlist mortality was higher in children < 2 years (HR 1.496) and in CHD (HR 1.702), while DCM was protective (HR 0.6312). Children < 2 years with diagnoses other than DCM had the highest waitlist mortality (HR 3.3). Recovery occurred in 7.8 %, mainly in infants < 1 year, with younger age and smaller height predicting improvement. ABO-incompatible transplantation was rare (8 cases) despite 278 (38.6 %) being <2 years at listing.

**Conclusion:**

Waitlist mortality among pediatric candidates in Germany remains high, particularly in young children with CHD. Broader application of ABO-incompatible transplantation and systematic evaluation for recovery may improve outcomes under persistent donor shortage.

## Introduction

Heart transplantation has become a standard of care for end-stage heart failure in pediatric patients. Survival rates among children awaiting heart transplantation have continuously improved, primarily due to the expanded use and enhanced management of mechanical circulatory support, particularly ventricular assist devices and modification in the allocation policy.^1^, ^2^ However, waitlist mortality and clinical deterioration represent persistent and significant obstacles in the management of pediatric patients awaiting heart transplantation.^3^, ^4^ Data on recovery rates among pediatric candidates listed for heart transplantation are limited and infrequently reported. Both pediatric cardiac waitlist mortality and recovery rates exhibit regional variation, largely due to significant differences in donor organ availability and allocation policies.^2^, ^3^, ^5^, ^6^ Donor organ shortage is a significant and persistent challenge in Germany, which has one of the lowest per capita donor rates in Europe and North America directly affecting waiting time and waitlist mortality. In Germany, pediatric heart transplantation is performed within the Eurotransplant network, which coordinates donor organ allocation across eight European countries. Allocation is primarily based on medical urgency, blood group compatibility, donor–recipient size matching, waiting time, and expected ischemic time. Pediatric candidates may be listed under regular transplantable (T) status or High Urgency (HU) status; however, the majority of children awaiting transplantation are listed as HU. HU listing provides prioritization for donor organ allocation in patients considered at immediate risk of death without transplantation. Despite ongoing discussions regarding survival benefit–based allocation models, the current Eurotransplant heart allocation system remains largely urgency based. Within Eurotransplant, ABO-incompatible pediatric heart transplantation is used selectively in infants with low isohemagglutinin titers to expand donor availability. However, ABO-compatible allocation remains the primary allocation strategy, and ABO-incompatible allocation is generally considered only when no suitable compatible recipient can be identified or when substantial survival benefit is anticipated. Given the differences in donor organ availability and allocation policies, substantial variations in waiting times, waiting mortality, and recovery rates among children listed for heart transplantation in Germany compared to other European or North American countries are likely. Most available data on waitlist mortality and recovery rates for pediatric heart candidates from large registries predominantly reflect patient populations in the U.S.^3^, ^4^, ^7^ To date, outcome data for pediatric patients listed for heart transplantation in Germany are limited to single-center experiences, with no published multicenter or nationwide analyses available. Therefore, we aimed to investigate clinical outcomes of this population, with a focus on waitlist mortality, removal due to clinical deterioration and rates of recovery.

## Methods

### Study design

This retrospective cohort analysis utilizes data from the German Transplant Registry (GTR), encompassing all solid organ donors and recipients in Germany. The data were sourced from Eurotransplant, the German Organ Transplantation Foundation, and the Institute for Quality Insurance and Transparency in Healthcare, respectively. The GTR comprises two datasets: a retrospective dataset covering the period from January 1st 2006 to December 31st 2016, and a prospective dataset collected from January 1st 2017 to December 31st 2020. We identified all children < 18 years who were listed for heart transplantation in both datasets. We evaluated key demographic and clinical variables in relation to clinical outcomes. The study was conducted in accordance with the Declaration of Helsinki. Due to the retrospective nature and analysis of registry data, this study was deemed exempt from the Institutional Review Board.

### Data transformation and statistical analysis

All data management, cleaning, and transformation procedures were performed in RStudio (version 2022.07.1) using the tidyverse package suite. Raw data were obtained from multiple registry-derived CSV files containing demographic, clinical, listing, transplantation, and follow-up information. Individual files were merged using unique patient and transplant identifiers (Eurotransplant number) to generate a unified longitudinal dataset. Duplicate entries were removed, and data formats were standardized prior to statistical analysis. All patients < 18 years of age at the time of the listing that were listed for heart transplantation were extracted for further analysis. Descriptive analysis was performed with categorical variables presented as frequency (percent) and continuous variables as median with (interquartile range, IQR) or mean (standard deviation, SD). Categorical variables were compared using the two-tailed chi square test. Data distribution was visualized by histogram and normality was tested with the Shapiro-Wilk test. All variables displayed non-normal distribution. Mann Whitney U or Kruskal-Wallis test was performed for analyzing non-parametric unpaired continuous variables. Potential risk factors were assessed by univariable and multivariable logistic regression analysis. Predictors were handled without transformation. A p-value of < 0.05 was considered statistically significant. Data were analyzed using SPSS statistics (version 23, IBM Corp., Armonk, NY, USA) and plotted with GraphPad Prism (Version 10, GraphPad Software, San Diego, CA, USA).

## Results

### Baseline characteristics and outcomes of pediatric heart transplant candidates

**Figure 1:**
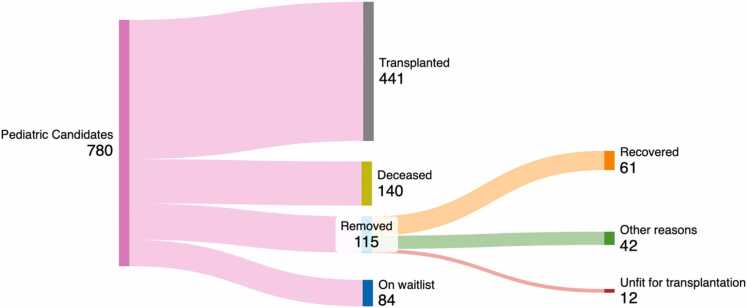
Outcomes of candidates listed for pediatric heart transplantation.

**Figure 2:**
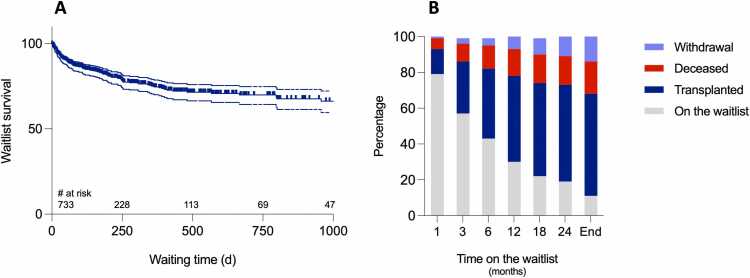
(A) Waitlist survival and (B) competing waitlist outcomes over time.

Characteristics of pediatric heart transplant candidates are summarized in Table 1. Among candidates male sex was more frequent with 56.7 %. The most reported diagnosis was dilated cardiomyopathy (DCM, 52.7 %), followed by congenital heart disease (CHD, 23.7 %). Median age at the time of listing was 5.74 years (IQR 0.70 – 14.06). The distribution of diagnoses across age categories is shown in Table 2. Median body weight was 18.1 kg (IQR 8–45) and median body height was 114 cm (IQR 74–157). The most frequent blood types were A (41.2 %) and O (39.7 %), 84.0 % were Rhesus positive. No differences were noted in baseline characteristics between the two eras. The nationality for listed pediatric candidates was German (72.4 %), from other European or non-European countries in 6 % and not listed for 21.5 %. Of the 780 listed pediatric candidates, 441 (56.5 %) underwent transplantation, 140 (17.9 %) deceased, 115 (14.7 %) were removed from the waitlist (61 for recovery, 12 for clinical deterioration, and 42 for undocumented reasons), and 84 (10.8 %) remained on the waitlist (Figure 1). Overall waitlist survival and competing waitlist outcomes over time are illustrated in Figure 2. The average waiting time for successfully transplanted candidates was 197 days (SD +/- 299, Table 1). We did not find significant differences in waiting time for children listed in the first era when compared to the more recent era. Candidates in the age groups 1–5 years (mean 244.6 +/-183.6 days) and 6–10 years (mean 235.4 +/-289.6 days) had significant longer waiting times compared to candidates < 1 year of age (mean 148.8 +/- 183.6 days) and candidates > 10 years (mean 185 +/-343 days, p = 0.016, Table 2).

**Table 1:** Transplant Candidates Listed in Germany in 2006 – 2020

N (%), Median (IQR)				
**Variable**	**All patients**	**2006–2016**	**2017–2020**	**P value**
	N = 780	N = 674	N = 106	
Sex				0.535
Female Male	338 (43.3 %) 442 (56.7 %)	292 (43.3 %) 382 (56.7 %)	46 (43.4 %) 60 (56.6 %)	
Age at listing (y)	5.74 (0.70–14.06)	5.54 (0.67–14.02)	6.62 (0.93–14.30)	0.381
− < 1 year − 1–5 years − 6–10 years − ≥ 11 years	222 (28.4 %) 174 (22.3 %) 99 (12.7 %) 284 (36.4 %)	195 (28.9 %) 150 (22.2 %) 83 (12.3 %) 245 (36.4 %)	27 (25.5 %) 24 (22.6 %) 16 (15.1 %) 39 (36.8 %)	
Body weight (kg)	18.1 (8−45)	17.6 (8−45)	23 (9.8–46.5)	0.206
Body height (cm)	114 (74−157)	112 (73−157)	122 (79−155)	0.378
ABO blood type				0.937
− A − B − AB − 0 − Rhesus +	321 (41.2 %) 103 (13.2 %) 46 (5.9 %) 310 (39.7 %) 655 (84.0 %)	279 (41.4 %) 87 (12.9 %) 40 (5.9 %) 268 (39.8 %) 569 (84.4 %)	42 (39.6 %) 16 (15.1 %) 6 (5.7 %) 42 (39.6 %) 86 (81.1 %)	
Diagnosis				0.731
− DCM − CHD − RCM − HCM − Graft dysfunction/retransplant listing − unknown	411 (52.7 %) 185 (23.7 %) 54 (6.9 %) 26 (3.3 %) 8 (1.0 %) 97 (12.4 %)	356 (52.8 %) 155 (23.0 %) 48 (7.1 %) 19 (2.8 %) 7 (1.0 %) 70 (10.4 %)	55 (51.9 %) 30 (28.3 %) 6 (5.7 %) 7 (6.6 %) 1 (0.9 %) 6 (5.7 %)	
Recipient nationality				**< 0.001**
− German − Other European − Non-European − unknown	565 (72.4 %) 14 (1.8 %) 33 (4.2 %) 168 (21.5 %)	465 (69.0 %) 14 (2.1 %) 26 (3.9 %) 169 (25.1 %)	100 (94.3 %) 4 (3.8 %) 2 (1.9 %) 0 (0 %)	
Listing outcome				**< 0.001**
− Transplanted − Death − Withdrawal − On list	441 (56.5 %) 140 (17.9 %) 115 (14.7 %) 84 (10.8 %)	382 (56.7 %) 126 (18.7 %) 107 (15.9 %) 59 (8.8 %)	59 (55.7 %) 14 (13.2 %) 8 (7.5 %) 25 (23.6 %)	
Waiting time (mean, +/- SD)	197 (+/- 299)	200 (+/- 300)	172 (+/- 247)	0.235
Recovered candidates	61 (7.8 %)	57 (8.5 %)	4 (3.7 %)	0.095
Cause of death				0.192
− Cardiac − Sepsis/MOF − Cerebrovascular − Hemorrhage/Embolic − unknown	53 (37.9 %) 30 (30 %) 12 (8.6 %) 7 (5 %) 38 (27.1 %)	47 (37.3 %) 29 (23.0 %) 10 (7.9 %) 3 (2.4 %) 33 (26.2 %)	6 (42.9 %) 1 (7.1 %) 2 (14.3 %) 2 (14.3 %) 3 (21.4 %)	
Time of death after listing (d)	58 (17−187)	58 (17−193)	58 (15−211)	0.430
Deceased or removed because unfit for transplantation	152 (19.5 %)	137 (20.3 %)	15 (14.2 %)	0.084
− deceased − removed	140 (17.9 %) 12 (1.5 %)	126 (18.7 %) 11 (1.6. %)	14 (14.1 %) 1 (0.9 %)	

**CHD** – Congenital heart disease, **DCM** – Dilated cardiomyopathy, **HCM** – Hypertrophic cardiomyopathy, **MOF** – Multi organ failure, **RCM** – Restrictive cardiomyopathy

**Table 2:** Diagnosis and Waiting Time According to Age Category

**Variable**	**< 1 year**	**1–5 years**	**6–10 years**	**> 10 years**	**P value**
	N=222	N=174	N=99	N=285	
Diagnosis					**<0.001**
− DCM − CHD − HCM − RCM − Graft dysfunction/retransplant listing − Other/Unknown	120 (54.1 %) 66 (29.7 %) 6 (2.7 %) 6 (2.7 %) 0 (0 %) 24 (10.8 %)	83 (47.7 %) 50 (28.7 %) 17 (9.8 %) 2 (1.1 %) 0 (0 %) 22 (12.7 %)	43 (43.4 %) 22 (22.2 %) 12 (12.1 %) 5 (5.1 %) 0 (0 %) 17 (17.1 %)	165 (57.9 %) 47 (16.5 %) 20 (7.0 %) 12 (4.2 %) 6 (2.1 %) 35 (12.2 %)	
Waiting time (d)					**0.016**
− Mean − SD	148.8 183.6	244.6 289.6	235.4 289.6	185.2 343.0	

**CHD** – Congenital heart disease, **DCM** – Dilated cardiomyopathy, **HCM** – Hypertrophic cardiomyopathy, **RCM** – Restrictive cardiomyopathy

### Waitlist mortality or removal for clinical deterioration

**Figure 3:**
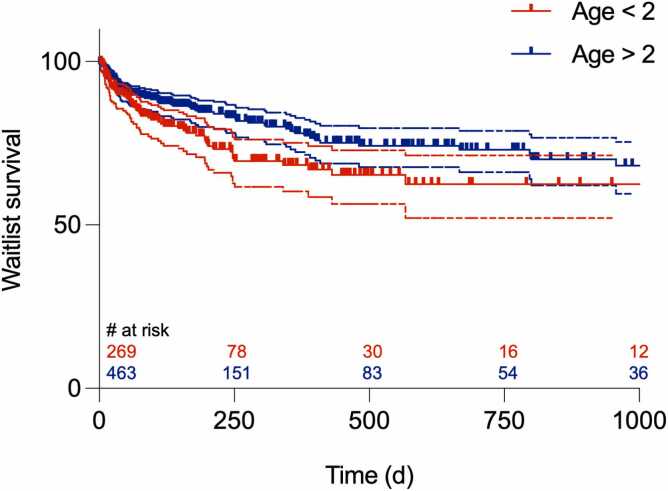
Candidates younger than 2 years of age at the time of the listing experienced significantly higher waitlist mortality (log rank 0.026, HR 1.496, 95% CI 1.059–2.114).

**Figure 4:**
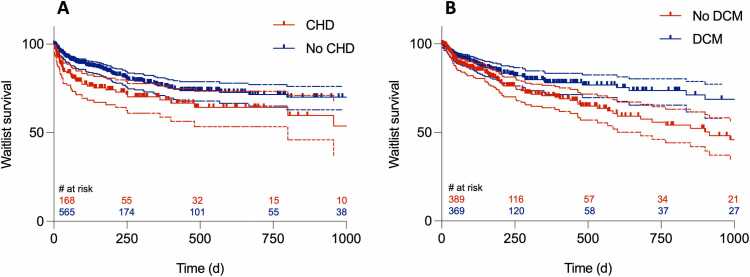
Waitlist mortality was significantly higher in (A) candidates with congenital heart disease (CHD, log rank p = 0.003, HR 1.702, 95%CI 1.144–2.534) and significantly lower in (B) in candidates with dilated cardiomyopathy (DCM, log rank p = 0.0061, HR 0.6312, 95% CI 0.4545–0.8767).

**Figure 5:**
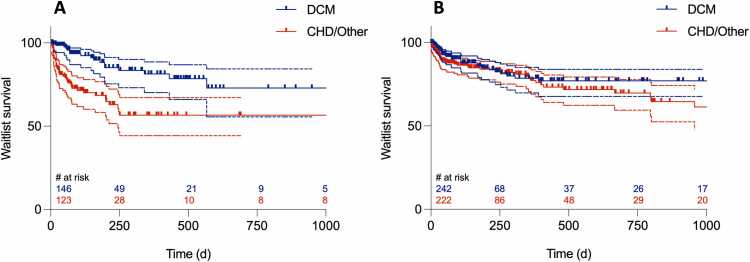
(A) Among candidates younger than 2 years of age at listing, waitlist mortality was significantly higher those with congenital heart disease (CHD) or other diagnoses compared with those with dilated cardiomyopathy (DCM, log rank p < 0.0001, HR 3.19, 95%CI 1.907–5.336). (B) Among candidates older than 2 years at the time of the listing, waitlist mortality was comparable between those with dilated cardiomyoapathy (DCM) and congenital heart disease (CHD) or other diagnoses (log rank p = 0.0914).

Of the pediatric candidates listed for heart transplantation, 152 (19.5 %) deceased (N = 140, 17.9 %) on the waitlist or were removed because they were unfit for transplantation (N = 12, 1.5 %, Table 1). The median time of death after listing was 58 days (IQR 17–187). The most common causes of death were cardiac (37.9 %), sepsis with multi-organ failure (MOF, 30 %), cerebrovascular (8.6 %) and hemorrhage/embolic (5 %). Cause of death was unknown in 27.1 % of the deceased candidates. Characteristics of candidates that deceased or were removed from the waitlist for clinical deterioration in comparison with other candidates are listed in Table 3. There were no differences in sex or transplant era between deceased/removed candidates and other. Deceased/removed candidates were significantly younger (median age 3.12 years, IQR 0.40 – 12.40) compared to other candidates (median age 6.44 years, IQR 0.87–14.29, p = 0.005). Sixty-nine percent of the deceased/removed candidates were ≤ 5 years of age. Deceased/removed candidates had a lower body weight (p < 0.001) and a smaller body size (p < 0.001) compared to other candidates. Deceased/removed candidates were less likely to have DCM (34.9 % vs. 57 %) and more likely to have CHD (32.2 % vs. 21.3 %, p < 0.001) compared to other candidates. Survival on the waitlist, assessed by Kaplan Meier analysis, was significantly lower among candidates younger than two years of age at the time of the listing (Figure 3, log rank p = 0.026, HR 1.496, 95% CI 1.059–2.114) and among children with CHD (Figure 4A, log rank p = 0.003, HR 1.707, 95%CI 1.198–2.432). In contrast, candidates with DCM demonstrated significantly better waitlist survival (Figure 4B, log rank p < 0.001, HR 0.505, 95%CI 0.357–0.715). Significant risk factors in univariable analysis were included in a multivariable Cox regression model. As age, body weight and height are colinear, only age was included. In multivariable analysis, younger age (HR 0.970, 95%CI 0.945–0.977, p < 0.001) was identified as a risk factor for death on or removal of the waitlist for clinical deterioration, whereas the diagnosis DCM reduced the risk (OR 0.546, 95 % CI 0.363–0.821, p = 0.041, Table 4). As candidates younger than 2 years of age experienced significant higher waitlist mortality than candidates older than 2 years, we further analyzed diagnosis-specific outcomes. Among candidates younger than 2 years of age at listing, waitlist mortality was significantly higher those with CHD/other diagnoses compared with those with DCM (log rank p < 0.0001, HR 3.19, 95%CI 1.907–5.336, Figure 5A). Among candidates older than 2 years at the time of the listing, waitlist mortality was comparable between those with DCM and CHD/other diagnoses (log rank p = 0.0914, Figure 5B).

**Table 3:** Candidates Deceased or Removed Because Unfit for Transplantation

N (%), Median (IQR)				
**Variable**	**All patients**	**Deceased/removed**	**Other**	**P-Value**
	N = 780	N = 152	N = 628	
Transplant era				0.084
− 2006–2016 − 2017–2020	674 (86.4 %) 106 (13.6 %)	137 (90.1 %) 15 (9.9 %)	537 (85.5 %) 91 (14.5 %)	
Sex				0.382
− Female − Male	338 (43.3 %) 442 (56.7 %)	68 (44.7 %) 84 (55.3 %)	270 (43.0 %) 358 (57.0 %)	
Age at listing (years)	5.74 (0.70–14.06)	3.12 (0.40–12.40)	6.44 (0.87–14.29)	**< 0.001**
− < 1 year − 1–5 years − 6–10 years − ≥ 11 years	222 (28.4 %) 174 (22.3 %) 99 (12.7 %) 284 (36.4 %)	56 (36.8 %) 34 (32.3 %) 17 (11.2 %) 45 (29.6 %)	166 (26.4 %) 140 (22.3 %) 82 (13.1 %) 239 (38.1 %)	
Body weight (kg)	18.1 (8−45)	11.2 (5.85–35.95)	20.65 (8.6–48.0)	**< 0.001**
Body height (cm)	114 (74−157)	89.5 (60−143)	120.5 (77−159)	**< 0.001**
Diagnosis				**< 0.001**
− DCM − CHD − RCM − HCM − Graft dysfunction/retransplant listing	411 (52.7 %) 185 (23.7 %) 54 (6.9 %) 26 (3.3 %) 12 (1.0 %)	53 (34.9 %) 49 (32.2 %) 10 (6.6 %) 5 (3.3 %) 8 (5.3 %)	358 (57 %) 134 (21.3 %) 44 (7.0 %) 21 (3.3 %) 4 (0.6 %)	
− Other − unknown	21 (2.7 %) 76 (9.7 %)	1 (0.7 %) 26 (17.1 %)	19 (3.0 %) 50 (8.0 %)	

**CHD** – Congenital heart disease, **DCM** – Dilated cardiomyopathy, **HCM** – Hypertrophic cardiomyopathy, **RCM** – Restrictive cardiomyopathy

**Table 4:** Univariable and Multivariable Cox Regression Analysis of Risk Factors for Death on the Waitlist

	**Univariable Cox regression analysis**			**Multivariable Cox regression analysis**		
**Variable**	**HR**	**95 % CI**	**P-Value**	**HR**	**95 % CI**	**P-Value**
Age	0.969	0.943–0.995	**0.02**	0.970	0.945–0.997	**0.03**
Body weight	0.985	0.977–0.993	**<0.001**			
Body height	0.992	0.987–0.996	**<0.001**			
Era 1	1.152	0.663–2.002	0.616			
DCM	0.505	0.357–0.715	**<0.001**	0.546	0.363–0.821	**0.004**
CHD	1.707	1.198–2.432	**0.003**	1.148	0.756–1.743	0.516

**CHD** – Congenital heart disease, **DCM** – Dilated cardiomyopathy, **HR** – Hazard ratio

### Subgroup analysis potential ABO incompatible candidates

Of the 780 listed candidates, 278 (38.6 %) were younger than two years at the time of the listing. Among these, 69 (24.8 %) died or were removed for clinical deterioration, 123 (44.2 %) underwent heart transplantation, and 86 (30.9 %) were still listed or removed following recovery. Of the 123 transplanted candidates, 12 were listed before the age of two but transplanted after turning two while 109 underwent transplantation within their first two years of life; for two candidates, age at transplantation was not available. Among the 109 recipients transplanted before two years of age, eight (7.3 %) received an ABO incompatible transplant, 84 (77.1 %) were ABO compatible, and in 17 cases (15.6 %) either donor or recipient blood type data was missing.

### Recovery on the waitlist and long-term outcomes

**Figure 6:**
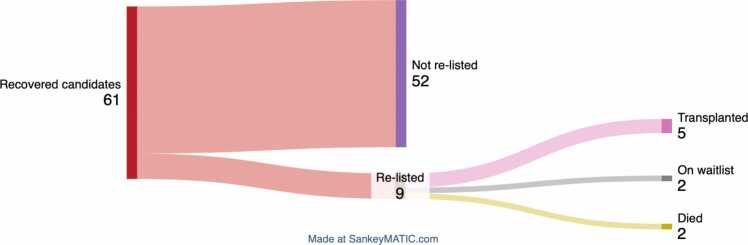
Outcomes of patients that were delisted due to recovery.

Of the 780 listed pediatric candidates, 61 (7.8 %) were removed from the waitlist for recovery (Table 1). Characteristics of candidates that recovered compared to other candidates are summarized in Table 5. The percentage of females within the recovered candidates was higher (52.5 % compared to 42.5 % in candidates that did not recover and 43.3 % of females overall), however this difference was not statistically significant. Recovered candidates were significant younger (median age 0.93 years, IQR 0.22 – 14.2) compared to candidates that did not recover (median age 6.24 years, IQR 0.87 – 14.03, p = 0.002). More than half of the recovered patients (50.8 %) were less than 1 year of age compared to 26.6 % in the non-recovery candidate group (p < 0.001). Body height was significantly smaller in the recovery group with a median of 86 cm (IQR 61 – 156) compared to 116 cm (IQR 75 – 157, p = 0.029). Consistently, body weight was lower in the recovery group, though the difference was not statistically significant. The percentage of candidates with DCM (which includes candidates with myocarditis) tended to be higher in the recovery group (62.3 %) compared to the non-recovery group (51.9 %). In univariable regression analysis for predictors of recovery were younger age (OR 0.948, 95 % CI 0.913–0.995, p = 0.018) and smaller body height (OR 0.994, 95% CI 0.987–1.000, p = 0.042, Table 6). Of the 61 recovered candidates 52 (85.2 %) were not listed again for heart transplantation, whereas 9 (14.8 %) were re-listed (Figure 6). Candidates required re-listing after a median time of 4.35 years (IQR 3.12–6.02). Of the re-listed recovered candidates, five successfully underwent transplantation, two died while on the waitlist and two were still listed.

**Table 5:** Recovered Candidates vs. Other

N (%), Median (IQR)				
**Variable**	**All patients**	**Recovery**	**No recovery**	**P-Value**
	N = 780	N = 61	N = 719	
Era				0.095
− 2006–2016 − 2017–2020	674 (86.4 %) 106 (13.6 %)	57 (8.5 %) 4 (3.7 %)	617 (91.5 %) 102 (96.2 %)	
Sex				0.134
− Female − Male	338 (43.3 %) 442 (56.7 %)	32 (52.5 %) 29 (47.5 %)	306 (42.5 %) 413 (57.4 %)	
Age at listing (years)	5.74 (0.70–14.06)	0.93 (0.22–14.2)	6.24 (0.87–14.03)	**0.002**
− < 1 year − 1–5 years − 6–10 years − ≥ 11 years	222 (28.4 %) 174 (22.3 %) 99 (12.7 %) 284 (36.4 %)	31 (50.8 %) 10 (16.4 %) 2 (3.3 %) 18 (29.5 %)	191 (26.6 %) 164 (22.8 %) 97 (13.5 %) 267 (37.1 %)	**< 0.001**
Body weight (kg)	18.1 (8−45)	10.0 (5.9–46.5)	19 (8.2–45)	0.065
Body height (cm)	114 (74−157)	82 (61−156)	116 (75−157)	**0.029**
Diagnosis				0.461
− DCM − CHD − RCM − HCM − Graft dysfunction/retransplant listing − Other/unknown	411 (52.7 %) 185 (23.7 %) 54 (6.9 %) 26 (3.3 %) 12 (1.0 %) 97 (12.4 %)	38 (62.3 %) 13 (21.3 %) 1 (1.6 %) 2 (3.3 %) 0 (0 %) 7 (11.5 %)	373 (51.9 %) 172 (23.9 %) 54 (7.5 %) 23 (3.2 %) 12 (1.7 %) 90 (12.5 %)	

**CHD** – Congenital heart disease, **DCM** – Dilated cardiomyopathy, **HCM** – Hypertrophic cardiomyopathy, **RCM** – Restrictive cardiomyopathy

**Table 6:** Univariable Regression Analysis for Predictors of Recovery

**Variable**	**Odds Ratio**	**95 % CI**	**P-value**
Female sex	1.489	0.822–2.515	0.136
Age	0.948	0.913–0.995	**0.018**
Body weight	0.994	0.982–1.005	0.288
Body height	0.994	0.987–1.000	**0.042**
DCM	1.550	0.905–2.654	0.111
CHD	0.926	0.463–1.653	0.680

**CHD** – Congenital heart disease, **DCM** – Dilated cardiomyopathy

### Outcomes of transplanted recipients

**Figure 7:**
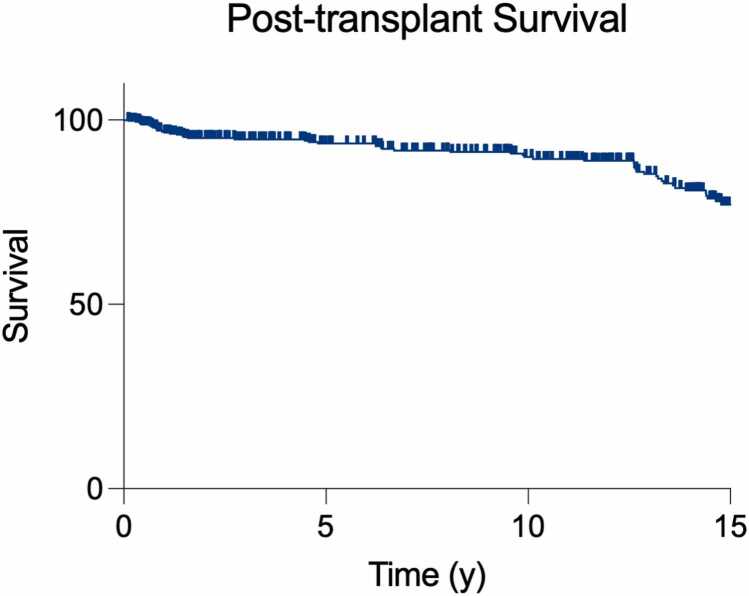
Post-transplant Kaplan-Meier survival analysis.

Data on post-transplant outcomes were available for 416 of 441 transplanted recipients (94.3%). The median follow-up after transplantation was 4.1 years (IQR 2.0–7.4) for the overall cohort, 4.2 years (IQR 2.0–7.6) for patients transplanted between 2006–2016, and 1.4 years (IQR 1.0–4.4) for those transplanted in the more recent era. Post-transplant survival estimated by Kaplan–Meier analysis is illustrated in Figure 7. Among patients who died after transplantation, the median time to death was 340 days (IQR 38–1691).

## Discussion

This study is the first multicenter registry analysis of outcomes of pediatric heart transplant candidates listed for heart transplantation in Germany. The most important findings are 1) while 56.5 % underwent heart transplantation, 17.9 % died on the waitlist and 1.4 % were removed for clinical deterioration. 2) Waitlist mortality significantly decreased from 18.7 in 2006–2016–13.2 % in 2017–2020 and was associated with younger age, lower body weight/height, and a diagnosis of CHD, whereas DCM was linked to lower risk. 3) Recovery occurred in 7.9 % of the candidates, primarily in infants under one year of age. 4) Despite nearly one-quarter of candidates being listed before the age of 2 years – the group with the highest waitlist mortality – ABO incompatible transplantation was rarely performed.

Overall wait-list mortality was high at 17.9 %, but decreased to 13.2 % in the more recent era. These findings are consistent with the results reported by Power et al., who analyzed United Network for Organ Sharing (UNOS) registry data and observed wait-list mortality of 17 % between 2006 and 2016 and 13 % between 2016 and 2023.^4^ Similarly, an analysis of the Pediatric Heart Transplant Society (PHTS) registry including 3928 candidates listed between 2010 and 2021 found a 12 % waitlist mortality; however, this analysis was limited to the first 12 months after listing.^8^ In contrast, registry data from Sweden reported a substantially lower waitlist mortality of 8.8 % between 2009 and 2023.^9^ Waitlist mortality was associated with younger age, smaller body size, and the diagnosis of CHD, whereas DCM was protective. Both younger age and CHD are well-recognized risk factors for waitlist mortality, as consistently demonstrated by analyses of the PHTS and Organ Procurement and Transplantation Network (OPTN) and single center experiences.^7^, ^8^, ^10^ In our registry cohort, children listed before the age of 2 years experienced the highest mortality. Further subgroup analysis revealed that this risk was driven by infants with CHD, who had significantly worse outcomes compared to those with DCM. In fact, waitlist survival did not differ between children listed before 2 years of age with DCM and those > 2 years of age with any diagnosis, underscoring that infants with CHD represent the group at greatest risk of death while awaiting transplantation. This increased vulnerability may be attributed to the more limited availability and consideration of VAD support as bridge to transplantation in young children with CHD, compared to those diagnosed with DCM.

In this registry, only 56.5 % of the listed candidates ultimately underwent heart transplantation, a proportion substantially lower than that reported in other large registries. For comparison, 70 % of the candidates listed in the UNOS registry between 2006 and 2023 underwent transplantation, while the PHTS reported a similar rate of 70 % censored at 12 months post listing between 2010 and 2021. ^4,8 4,8^Likewise, the Swedish national registry documented a transplantation rate of 72.8 %. ^9^ In the German cohort, 19.5% of the patients died or were removed from the waitlist because of clinical deterioration, compared with 16% in the UNOS registry and 15% in the PHTS registry (censored at 12 months). In UNOS, 8% of patients recovered and were delisted, and 4% remained listed. In PHTS, 16% remained listed and no patients were delisted for recovery, likely reflecting the 12-month censoring. 4, 8 In our cohort, 7.8% were delisted due to recovery, 10.8% remained listed, and 5.4% were delisted for undocumented reasons. Overall, transplantation rates were lower, while waitlist mortality or delisting for clinical deterioration or unknown reasons was higher. Recovery rates were comparable to UNOS, and a greater proportion of patients remained listed long term. This might reflect a comparably greater donor organ shortage in Germany relative to other countries. Consistent with these findings, the average waiting time for transplanted candidates in our cohort was 197 days, notably longer than those reported elsewhere. The UNOS registry documented a mean waitlist duration of 180 days in 2006–2016, which declined to 152 days in 2016–2023.^4^ In Sweden, the multicenter analysis reported a median waiting time of 79 days (range 1–186) between 2009 and 2023, noting a significant increase compared with the preceding era. ^9^ In our cohort, although the difference did not reach statistical significance, waiting times appeared shorter in the more recent era, with an average of 172 days.

In our cohort, 278 of 780 candidates (36 %) were younger than 2 years of age at the time of the listing; however, only 8 candidates (1.0 % of all listed candidates, 2.9 % of all listed candidates < 2 years) underwent ABO incompatible transplantation. Notably, this age group demonstrated the highest mortality on the waitlist, underscoring a potentially modifiable risk that could be addressed through changes in allocation policy. Data from the United States support this: following policy changes in 2016 that recipients < 2 years of age with ABO compatible or incompatible listing were given equal priority, UNOS reported a 27 % increase in ABO-incompatible transplants which was accompanied by reduction in mortality among infants.^4^, ^11^ After the policy change, 65 % of all children < 2 years of age were listed and 30 % transplanted ABO incompatible in the UNOS registry.^11^ Similar to our data, in Sweden ABO incompatible transplantation accounted for only 8.0 % of pediatric heart transplants between 2009 and 2023, and was preferentially utilized in patients with high immunologic risk. ^9^ Notably, the German transplant registry data set did not provide the information, if a candidate was listed ABO incompatible, we could just extract ABO incompatible transplantations. Moreover, there were missing information about donor blood type in 61 of 416 transplanted cases (14.6 %), so the actual rate might have been slightly higher. However, if only the 355 cases with eligible information were included in the analysis, the percentage of ABO incompatible transplantations would remain low with 2.3 %. Despite increasing experience with ABO-incompatible pediatric heart transplantation internationally, its utilization within the Eurotransplant region remains limited because ABOi allocation is currently restricted to selected children younger than 2 years of age with low isohemagglutinin titers. As donor availability is particularly limited in infants and small children, restricted access to ABOi transplantation may contribute to prolonged waiting times and persistently elevated waitlist mortality in this vulnerable population. Given the substantial survival benefit associated with timely transplantation in critically ill pediatric candidates, broader consideration of ABOi allocation strategies and further evaluation of current Eurotransplant policies may help to improve donor access and reduce waitlist mortality, particularly among young children with advanced heart failure or complex congenital heart disease.^12^

Data on recovery among pediatric candidates listed for heart transplantation remain scarce. In our registry analysis, the recovery rate was 7.8 %. Similarly, the UNOS registry reported that 8 % of candidates between 2006 and 2016, and 2.6 % between 2017 and 2023, were reported as „improved“.^4^ Recovery leading to withdrawal from the wait list was observed predominantly in infants, with more than half being younger than 1 year at the time of listing. Younger age and smaller body size emerged as significant predictors of recovery, consistent with factors previously identified following durable VAD support.^13^, ^14^ Given the persistent shortage of donor organs, systematic evaluation of recovery potential in listed candidates warrants further study, and standardized protocols for such assessments are needed.

## Conclusion

This first multicenter registry analysis of pediatric heart transplant candidates in Germany provides important insights into outcomes and risk factors affecting this vulnerable population. Waitlist mortality remained high though it has declined in recent years, aligning with international trends. Younger age, smaller body size, and a diagnosis of congenital heart disease (CHD) were associated with increased mortality, highlighting infants with CHD as the highest-risk group. In contrast, children with dilated cardiomyopathy demonstrated better waitlist survival. Transplantation rates were substantially lower than rates reported in other registries – suggesting a relative shortage of donor organs in Germany which was also reflected in longer waiting times. Despite the high mortality among infants, ABO-incompatible transplantation, a proven strategy to expand the donor pool for this group, was rarely utilized. Expanding its use could represent a feasible policy intervention to improve outcomes in infants under two years of age. Recovery from end-stage heart failure occurred in a small but notable proportion of listed candidates, particularly among infants, emphasizing the importance of systematic evaluation and standardized criteria for identifying patients with potential recovery.

## Limitations

The study is retrospective and relies entirely on registry data, which limits its ability to establish causality. Data quality depends on the accuracy and completeness of reporting by participating centers and the registry, introducing potential reporting bias or data entry errors. Missing or incomplete (follow-up) data may affect the reliability of outcome estimates. Furthermore, the registry lacks detailed clinical variables, such as information on mechanical circulatory support or details on the cardiac diagnosis. The two registry eras differ in data collection methods and completeness resulting in non-uniform data sets. These limitations should be considered when interpreting the observed associations.

## Disclosure statement

No funding was received for this study. The authors declare that they have no known competing financial interests or personal relationships that could have appeared to influence the work reported in this paper.
